# Etymologia: influenza

**DOI:** 10.3201/eid1201.ET1201

**Published:** 2006-01

**Keywords:** etymology, etomologia, influenza

## [in′′floo-en′zə]

Acute viral infection of the respiratory tract. From Latin *influentia*, “to flow into”; in medieval times, intangible fluid given off by stars was believed to affect humans. The Italian *influenza* referred to any disease outbreak thought to be influenced by stars. In 1743, what Italians called an *influenza di catarro* (“epidemic of catarrh”) spread across Europe, and the disease came to be known in English as simply “influenza.”

**Sources:** Dorland’s illustrated medical dictionary. 30th ed. Philadelphia: Saunders; 2003 and Quinion M. World wide words. 1998 Jan 3 [cited 2005 Dec 5]. Available from http://www.worldwidewords.org/topicalwords/tw-inf1.htm

